# The *APETALA2* homolog *CaFFN* regulates flowering time in pepper

**DOI:** 10.1038/s41438-021-00643-7

**Published:** 2021-11-01

**Authors:** Xinjie Yuan, Rong Fang, Kunhua Zhou, Yueqin Huang, Gang Lei, Xiaowu Wang, Xuejun Chen

**Affiliations:** 1 Institute of Vegetables and Flowers, Jiangxi Academy of Agricultural Sciences, 330200 Nanchang, China; 2 Institute of Vegetables and Flowers, Chinese Academy of Agricultural Sciences, 100081 Beijing, China

**Keywords:** Natural variation in plants, Agricultural genetics

## Abstract

Flowering time is an important agronomic trait that contributes to fitness in plants. However, the genetic basis of flowering time has not been extensively studied in pepper. To understand the genetics underlying flowering time, we constructed an F_2_ population by crossing a spontaneous early flowering mutant and a late-flowering pepper line. Using bulked segregant RNA-seq, a major locus controlling flowering time in this population was mapped to the end of chromosome 2. An *APETALA2* (*AP2*) homolog (*CaFFN*) cosegregated with flowering time in 297 individuals of the F_2_ population. A comparison between the parents revealed a naturally occurring rare SNP (SNP2T > C) that resulted in the loss of a start codon in *CaFFN* in the early flowering mutant. Transgenic *Nicotiana benthamiana* plants with high *CaFFN* expression exhibited a delay in flowering time and floral patterning defects. On the other hand, pepper plants with *CaFFN* silencing flowered early. Therefore, the *CaFFN* gene acts as a flowering repressor in pepper. CaFFN may function as a transcriptional activator to activate the expression of *CaAGL15* and *miR156e* and as a transcriptional repressor to repress the expression of *CaAG*, *CaAP1*, *CaSEP3*, *CaSOC1*, and *miR172b* based on a qRT-PCR assay. Direct activation of *CaAGL15* by CaFFN was detected using yeast one-hybrid and dual-luciferase reporter assays, consistent with the hypothesis that CaFFN regulates flowering time. Moreover, the *CaFFN* gene association analysis revealed a significant association with flowering time in a natural pepper population, indicating that the *CaFFN* gene has a broad effect on flowering time in pepper. Finally, the phylogeny, evolutionary expansion and expression patterns of *CaFFN/AP2* homologs were analyzed to provide valuable insight into *CaFFN*. This study increases our understanding of the involvement of *CaFFN* in controlling flowering time in pepper, thus making *CaFFN* a target gene for breeding early maturing pepper.

## Introduction

The timing of the transition from vegetative growth to flowering is a major factor that ensures that plants produce enough progeny for their sustainable development^1–3^. Thus, flowering time is an important agronomic trait that contributes to fitness in annual plants. Genetic analysis of the model plant *Arabidopsis thaliana* has identified numerous genes involved in flowering-time control that may participate in the photoperiod, vernalization, ambient temperature, aging, autonomous or gibberellin pathways^4^, each of which plays either a floral inhibiting, neutral, or promoting function in response to exogenous or endogenous signals. Downstream of these pathways are a small number of floral integrator genes, including *FLOWERING LOCUS T* (*FT*) and *SUPPRESSOR OF OVEREXPRESSION OF CONSTANS 1* (*SOC1*)^5–9^. ‘Integrators’ can activate floral meristem identity (FMI) genes, such as *LEAFY* (*LFY*), *APETALA1* (*AP1*), *APETALA2* (*AP2*), *FRUITFULL* (*FUL*), and *CAULIFLOWER* (*CAL*), which encode proteins that promote floral development^1^. Among these FMI genes, the transcription factor AP2 directly promotes the expression of the floral repressor *AGAMOUS-LIKE15* (*AGL15*) and directly represses the expression of the flowering promoting transcripts *SOC1*, *AP1*, *SEPALLATA3* (*SEP3*), *AGAMOUS* (*AG*), and *FUL*^10^. In addition, AP2 positively regulates *miR156* and negatively regulates *miR172* to form a complex direct feedback loop that influences *AP2* expression^10^.

*Solanaceae* includes species such as potato (*Solanum tuberosum*), tomato (*S. lycopersicum*), pepper (*Capsicum annuum*), eggplant (*S. melongena*), and tobacco (*Nicotiana tabacum*) that are of agricultural interest or research interest. Among these species, tomato has been more extensively studied for the control of flowering time^11–13^. As a member of the *Solanaceae* family, pepper (*C*. *annuum* L.) has a sympodial shoot structure. The pepper shoot apical meristem (SAM) first produces stems and leaves alternately in the vegetative phase and then, upon the transition to the reproductive phase, terminates in an inflorescence meristem (IM) that subsequently develops into the first flower; plant growth continues from the upper most axillary meristem^14,15^. Flowering time in pepper can be best measured by counting the number of leaves (or nodes) on the primary stem (Nle) from the cotyledon until the first flower, which has also been described as the number of first flower nodes (FFNs)^16–18^.

In pepper, several genes controlling flowering time have been identified by screening an EMS-mutagenized population. Of these genes, mutants of *CaJOINTLESS* (*CaJ*), *CaBLIND* (*CaBL*) and *CaS* were late flowering^14,15,19^, and mutants of *fasciculate* (*fa*) and *E-62* were early flowering^20,21^. These genes have a pleiotropic effect on plant growth in addition to affecting flowering time in pepper; for example, *CaS* is required for flower formation, and *CaBL* regulates axillary meristem initiation^15,19^. However, most of these mutant genes are unlikely to be utilized in pepper breeding; previous studies have reported that mutants of *CaS* were characterized by complete inhibition of flower formation^15,19^, and mutants of *CaBL* had no axillary shoot development in the vegetative phase and only two sympodial units in the reproductive phase^19^.

Pepper germplasm resources exhibit extensive natural variation in flowering time^22^. Genetic analysis showed that the flowering time trait in pepper was a quantitative trait commonly controlled by a few major genes and some minor genes, as well as environmental factors such as temperature and photoperiod^16,17,23,24^. Quantitative trait loci (QTLs) affecting flowering time have been identified on different chromosomes using different populations^17,18,23–26^. For example, Tan et al.^17^ mapped one major and five minor QTLs affecting FFNs on chromosomes P2, P7, P10 and P11 based on an interspecific genetic map, and 12 genes were recommended as important candidate genes for the major QTL *Nle2.2*. Zhang et al.^18^ identified 23 candidate genes on chromosome 12 within an interval of 3.98 Mb using combined specific-locus amplified fragment sequencing (SLAF-seq) and bulked segregant analysis (BSA). Zhu et al.^26^ detected three flowering time QTLs based on an interspecific F_2_ population, in which an AP2 protein encoding gene *Capana02g000700* was predicted to be the candidate gene for the major QTL. However, further studies to identify and validate the function of candidate genes controlling flowering time in pepper have been limited to date.

The early flowering pepper B_9431_, which can blossom and bear fruit at the first node on the main stem, is an ideal natural material for determining the genetic control of flowering time in pepper. In this study, an F_2_ population with 297 plants derived from an intraspecific cross between inbred lines B_9431_ (early flowering) and A_145_ (late flowering) was developed. The locus controlling flowering time in this population was mapped by the bulked segregant RNA-Seq (BSR-Seq) method. The heterologous overexpression assay in *N*. *benthamiana* and the virus-induced gene silencing (VIGS) assay in pepper A_145_ were performed to verify the function of the candidate gene *CaFFN*. Similarly, a real-time fluorescent quantitative PCR assay, a yeast one-hybrid assay and a dual-luciferase reporter assay were conducted to determine how the *CaFFN* gene influences the flowering time of pepper. Furthermore, *CaFFN* gene association analysis was performed on 167 pepper inbred lines to test the effect of this gene on the control of natural variations in flowering time. The phylogeny, evolutionary expansion and expression patterns of *CaFFN/AP2* homologs were analyzed to provide useful insight into *CaFFN*.

## Results

### Genetic analysis of the flowering time trait in pepper

**Fig. 1:**
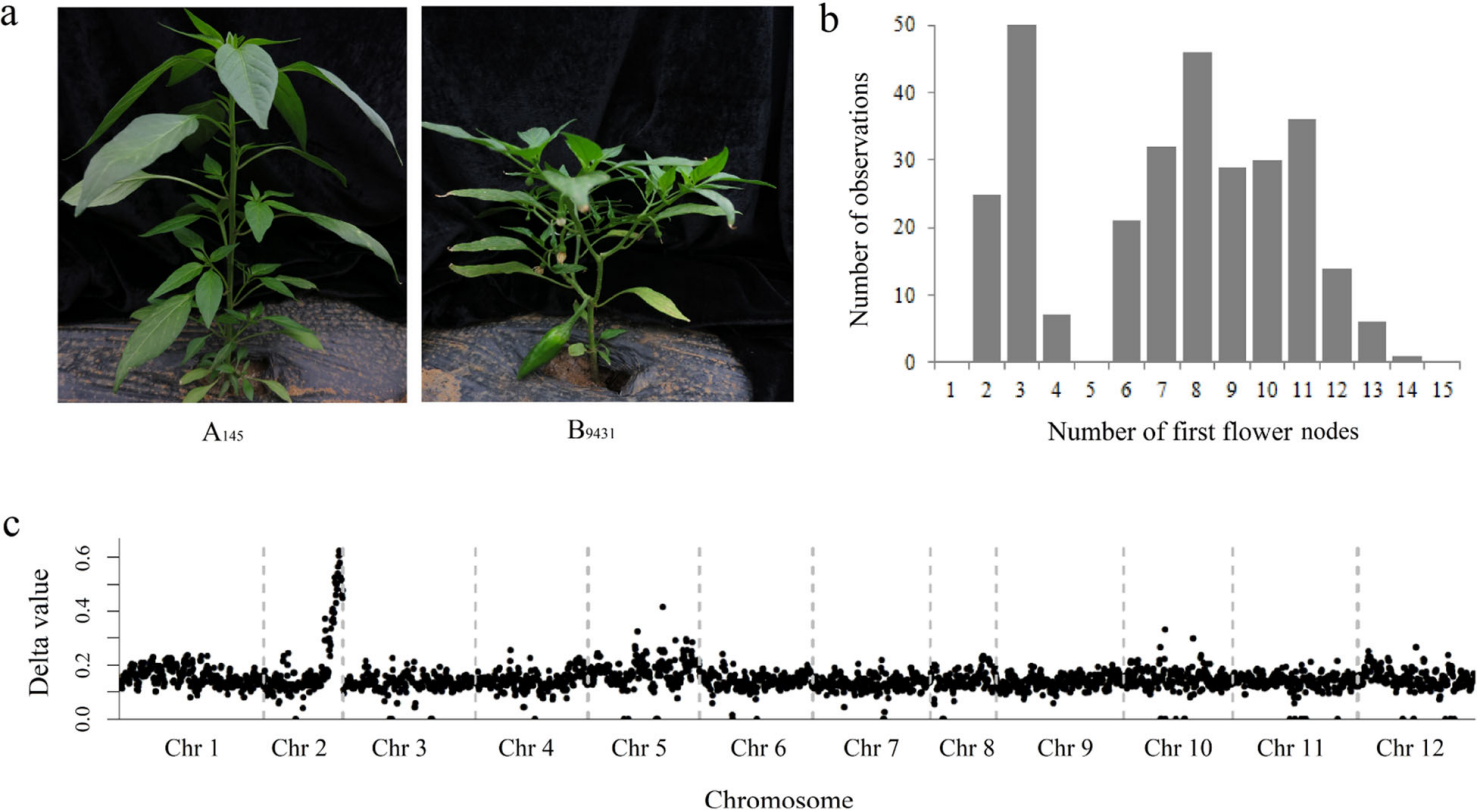
Genetic analysis of the flowering time trait in pepper. **a** Pictures of the late-flowering parent A_145_ and early flowering parent B_9431_. **b** Frequency distribution of flowering time (number of first flower nodes) in the F_2_ population. **c** Differences in SNP frequencies between the early and late-flowering pools from the F_2_ population. The horizontal axis shows the 12 chromosomes in pepper. The vertical coordinate represents the difference in SNP frequencies between the two pools. A peak SNP value at the end of chromosome 2 suggests that one strong major locus contributes to the variation in flowering time in this population

A natural precocious variant was discovered in our breeding work, from which a stable early flowering inbred line named B_9431_ was created after years of selfing and selective breeding (Fig. 1a). B_9431_ can flower after the development of one leaf on the primary stem. To investigate the inheritance of flowering time in pepper, an F_2_ population including 297 plants developed from a cross between B_9431_ and A_145_ was studied. Flowering time, in this study, was measured by counting the number of leaves on the primary stem between the cotyledon and the first flower (referred to as the number of FFNs) according to previous studies^16–18^. The average number of FFNs on parent B_9431_ was 2.3 (ranging from 1 to 4), parent A_145_ was 14 (ranging from 13 to 15), and the F_1_ progeny was 8.5 (ranging from 8 to 10); that on the F_2_ population ranged from 2 to 14. The F_2_ population showed a bimodal distribution (Fig. 1b). Based on a distribution of the F_2_ population at the low point (number of FFNs of 5), the proportion of late-flowering (number of FFNs ranging from 6 to 14) to early flowering (number of FFNs ranging from 2 to 4) peppers was ~3:1 (*χ*^*2*^ = 1.08; *p* = 0.30), thus indicating the presence of one strong major gene for this trait^27,28^.

### BSR-Seq identified the major locus underlying flowering time in pepper

Thirty plants with the minimum number of FFNs from the F_2_ population were chosen to construct an ‘early flowering pool’, and 30 plants with the maximum number of FFNs were chosen to construct a ‘late-flowering pool’. The total RNA of the two pools was extracted for Illumina high-throughput sequencing, and the clean data were aligned to the *C*. *annuum* cv. CM334 genome chromosomes (release 1.55) (http://peppergenome.snu.ac.kr/). The frequencies for the single-nucleotide polymorphisms (SNPs) thus detected were calculated for each pool. The average difference in the SNP frequency (index) between the two pools was calculated across a 1.0 Mb genomic interval using a 3.0 Mb sliding window, and the results were plotted against all twelve chromosomes of the reference genome (Fig. 1c). The figure shows an obvious peak in the SNP delta values at the distal end of chromosome 2, which indicates one strong major locus governing flowering time in this region.

### Identification of a candidate gene with a point mutation at the start codon in the early flowering parent B_9431_

**Fig. 2:**
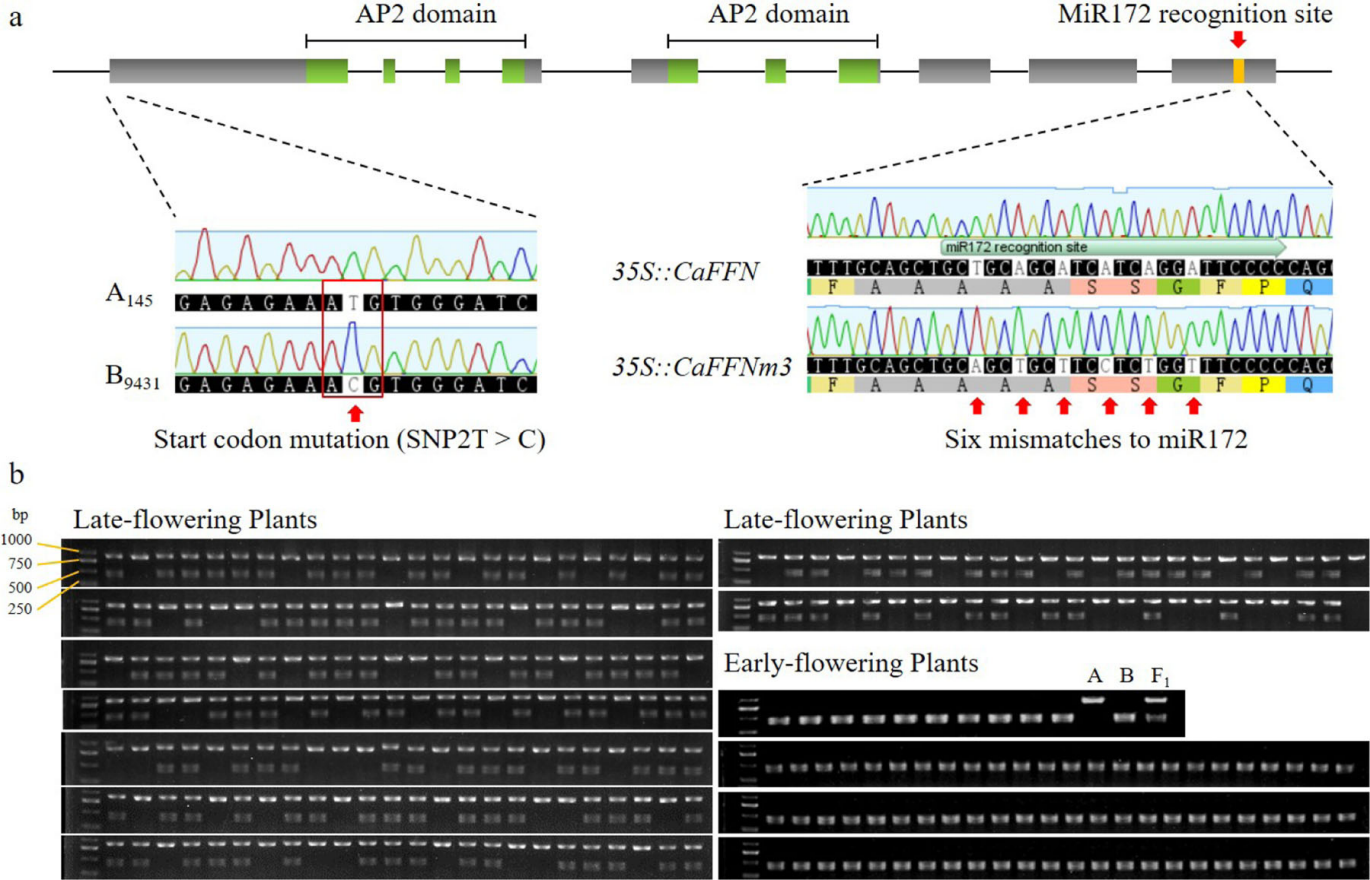
The genotypes of the *CaFFN* gene. **a** Schematic representation of the *CaFFN* gene. The start codon of the *CaFFN* gene in B_9431_ had a point mutation from T to C (SNP2T > C). The *miR172* recognition site in *35::CaFFNm3* had six mismatches to *miR172*. **b** Marker CSF2 identified SNP2T > C through the digestion of PCR products by the restriction enzyme *TaiI*. ‘A’ represents A_145_, ‘B’ represents B_9431_, ‘F_1_’ represents the F_1_ population of B_9431_ × A_145_, ‘Late-flowering plants’ represents individuals with a number of first-flower nodes (FFNs) from 6 to 14 in the F_2_ population, and ‘Early flowering plants’ represents individuals with a number of FFNs from 2 to 4 in the F_2_ population

At the end of chromosome 2, the AP2 transcription factor-encoding gene *CA02g14540* caught our attention, since its likely ortholog *AP2* in *Arabidopsis* has numerous functions, including participation in floral transition and development. The sequences of this gene in the two parents of the F_2_ population were determined by sequencing the PCR products amplified with the primers listed in Supplementary Table S1. A point mutation at the start codon ATG (referred to as SNP2T > C) was discovered in the early flowering parent B_9431_ (Fig. 2a). This SNP led to the loss of the original start codon of *CaFFN* and resulted in a putative frameshift mutation of *CaFFN* in B_9431_. To investigate the potential of this gene to be a candidate gene controlling flowering time in this study, a cleaved amplified polymorphic sequence (CAPS) marker CSF2 (Fig. 2b, Supplementary Table S1) was developed based on SNP2T > C. The 297 individuals in the F_2_ population of B_9431_ × A_145_ were genotyped using marker CSF2. The results showed that all early flowering (number of FFNs ranging from 2 to 4) plants had the same genotype as B_9431_, and all late-flowering (number of FFNs ranging from 6 to 14) plants had the same genotype as A_145_ or the F_1_ (Fig. 2b), indicating cosegregation of CSF2 with flowering time. These results demonstrated that *CA02g14540* was a candidate gene (referred to as the *CaFFN* gene) underlying flowering time in the F_2_ population of B_9431_ × A_145_.

### Two *N. benthamiana* transgenic lines expressing *CaFFN* exhibited a notable delay in flowering time

To investigate whether *CaFFN* might function in controlling flowering time, transgenic *N. benthamiana* lines expressing pepper *CaFFN* under the control of the CaMV 35S promoter were generated. However, no obvious changes in flowering time or flower patterning were observed in the 32 transgenic lines, even though the expression of the *CaFFN* gene was detected by qRT-PCR.

**Fig. 3:**
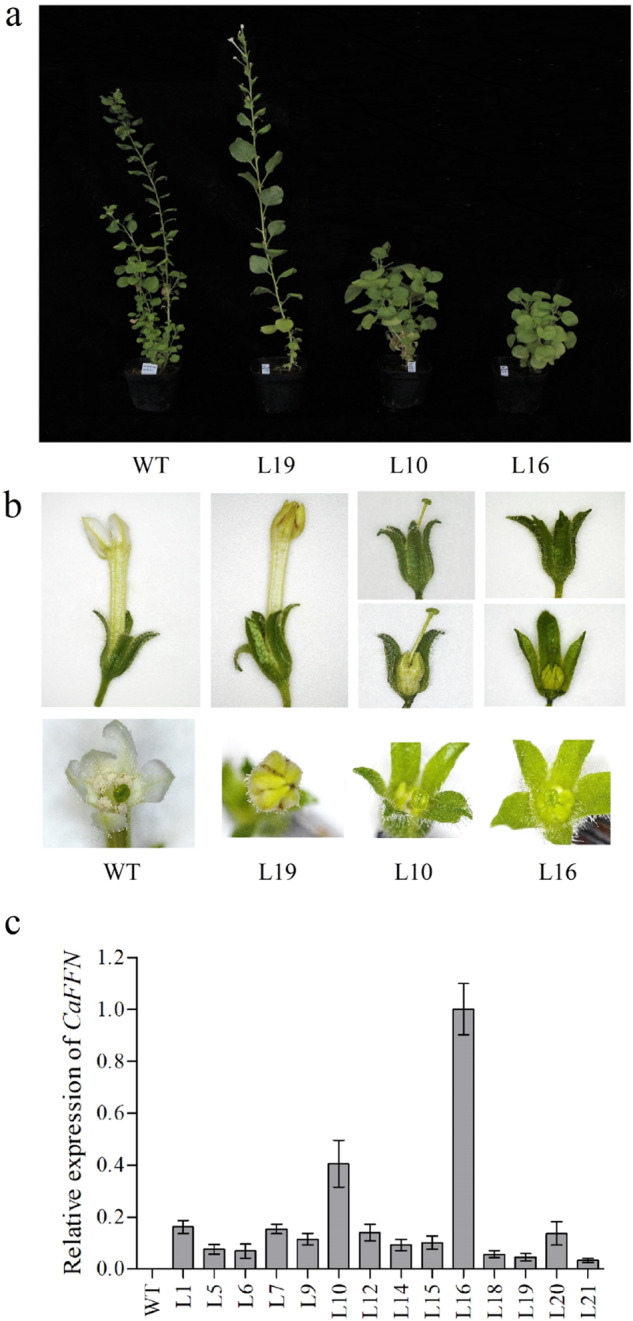
Phenotypes of *35S::CaFFNm3* lines and transcript levels of the *CaFFN* gene in transgenic lines. **a** *35S::CaFFNm3* line 10 (L10) and line 16 (L16) produced numerous leaves during a prolonged vegetative growth period and flowered later than the wild-type (WT) *Nicotiana benthamiana* and other *35S::CaFFNm3* lines, such as line 19 (L19). **b** Floral patterning changes in L10 and L16. L10 and L16 produced shorter filaments and poorly developed petals with a reduced size compared to the WT and other *35S::CaFFNm3* lines. Two sepals were removed to reveal the petals inside the L10/L16 flower (the second panel of L10/L16). Flowers were photographed 30 days post flowering. **c** Transcript levels of *CaFFN* in 14 transgenic lines and wild-type (WT) *N*. *benthamiana*. Error bars represent the standard deviation of three technical repeats

*CaFFN* is an *AP2*-like gene with a putative conserved *miR172* target site. It is thus speculated that a modification of the *miR172* target site would increase the expression of the *CaFFN* gene. For this reason, 22 transgenic lines transformed with modified *CaFFN* cDNA containing six mismatches (synonymous mutations) to *miR172* (*miR172*-resistant *CaFFN* mutant, *35S::CaFFNm3*) were generated (Fig. 2a). Among them, two transgenic lines (lines 10 and 16) exhibited a notable delay in flowering time (Fig. 3a).

### Levels of *CaFFN* mRNA were high in the late-flowering *35S::CaFFNm3* lines

To further explore why these two transgenic lines behaved differently, 14 transgenic lines transformed with *35S::CaFFNm3* were used for qRT-PCR analysis. Primers FFN-RT-jF4 and CaFFN-sNR2 (Supplementary Table S1) specific to *CaFFN* were designed to examine the expression level of *CaFFN* in *N*. *benthamiana*. The results revealed that the expression level of the *CaFFN* gene was higher in these two phenotypic lines than in other plants (Fig. 3c). These two transgenic lines produced numerous leaves in an extended period of vegetative growth and flowered two and a half months later than control plants. In addition to the delayed-flowering phenotype, sterility and floral patterning defects, which were characterized by shorter filaments and poorly developed petals with a reduced size, were observed in line 10 (L10) and line 16 (L16) (Fig. 3b). L16, with the highest level of *CaFFN* mRNA, exhibited the most dramatic change; it produced the smallest petals and an even shorter style (Fig. 3b). These results provide evidence that the *CaFFN* gene has a function in regulating flowering time and floral development.

### Silencing of the *CaFFN* gene promotes early flowering in pepper

**Fig. 4:**
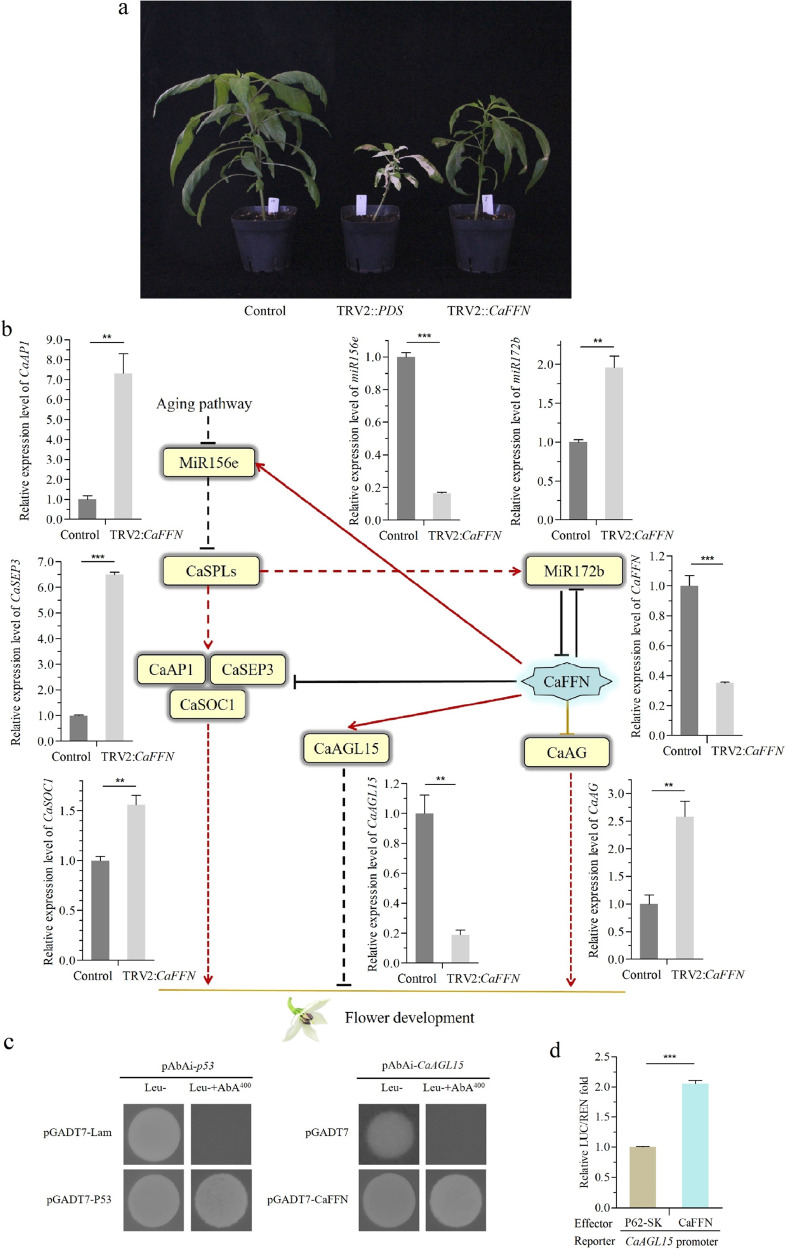
Functional analysis of the *CaFFN* gene in pepper. **a** *CaFFN* gene silencing in A_145_ via VIGS caused early flowering. TRV2::*PDS* plants showed photobleaching, and TRV2-*CaFFN* plants flowered earlier (the number of FFNs was 8 ± 0.30) than control plants (the average number of FFNs was 14 ± 0.26). **b** Relative gene expression levels in *CaFFN* gene-silenced pepper plants. The expression level of the *CaFFN* gene was reduced via VIGS compared to the control in the shoot apical meristem at the four FFN stage. *CaAG*, *CaAP1*, *CaSEP3*, *CaSOC1*, and *miR172b* were upregulated in *CaFFN*-silenced plants. *CaAGL15* and *miR156e* were downregulated in *CaFFN*-silenced plants. Error bars represent the standard error of three biological replicates. Asterisks show significant differences between TRV2::*CaFFN* and the control (Student’s *t* test, **p < 0.01, ***p < 0.001). Hypothetical models for the transcriptional regulatory network involving CaFFN according to the transcriptional relationships in this study and reports in *Arabidopsis*^10^. Arrows represent activation, and bars represent inhibition. **c** CaFFN bound to the cis-elements of *CaAGL15* in the yeast one-hybrid (Y1H) assay. pGADT7-p53/pAbAi-p53 and pGADT7-Lam/pAbAi-P53 were used as positive and negative controls, respectively. Superscripts represent the concentration of aureobasidin A (AbA). **d** The ability of CaFFN to bind to the promoter of *CaAGL15* was tested by dual luciferase assay. The LUC/REN fold value of the empty vector P62-SK plus promoter was set to 1.0. The values are the means ± SD (Student’s *t* test, ***p < 0.001)

To study the functional roles of *CaFFN* in flowering time in pepper, loss-of-function experiments were performed on parental line A_145_, in which *CaFFN* was silenced by VIGS. *Agrobacterium* carrying the tobacco rattle virus (TRV)-based vector TRV2::*CaFFN* mixed with *Agrobacterium* carrying the TRV1 vector were infiltrated into pepper seedlings at the four-leaf stage. Seedlings injected with TRV2::*PDS-* and TRV1-containing *Agrobacterium* were used as controls to measure the effect of gene silencing, which induced a photobleaching phenotype. Seedlings injected with TRV2- and TRV1-containing *Agrobacterium* were used as negative controls. The infiltrated seedlings were grown at 20 ± 1 °C and 90 ± 5% relative humidity to enhance silencing efficiency. Plants injected with TRV2::*PDS* showed photobleaching (Fig. 4a), suggesting successful gene silencing in pepper A_145_. Plants injected with TRV2::*CaFFN* flowered earlier (the average number of FFNs was eight, with a standard error of 0.30) than control plants (the average number of FFNs was 14, with a standard error of 0.26) (Fig. 4a). Consistently, qRT-PCR results showed an obvious decline in the expression level of *CaFFN* in TRV2::*CaFFN* plants compared to the control plants (Fig. 4b). The result that silencing of the *CaFFN* gene leads to early flowering in TRV2::*CaFFN* plants further shows the function of CaFFN as a flowering repressor in pepper.

### Gene expression changes in response to *CaFFN* silencing

The above results in this study indicated that CaFFN is a repressor of flowering. However, the mechanism underlying flowering time alteration caused by *CaFFN* remains to be determined. As the closest homolog to *AP2*, determining whether the network surrounding *AP2* is conserved and applicable to *CaFFN* is worthy of our attention. For this purpose, the likely orthologs of *AG*, *AGL15*, *AP1*, *SEP3*, and *SOC1* (*CaAG* [*CA02g12980*], *CaAGL15* [*CA01g21270*], *CaAP1* [*CA02g27000*], *CaSEP3* [*CA11g09730*], and *CaSOC1* [*CA01g19500*]) were searched by reciprocal best-hit analysis in the *C*. *annuum* cv. CM334 genome (release 1.55) (http://peppergenome.snu.ac.kr/). The expression levels of these flowering time and floral organ development genes were analyzed by qRT-PCR relative to the housekeeping gene *CaGAPDH* [*CA03g24310*]. The results showed that the expression levels of *CaAG*, *CaAP1*, *CaSEP3*, and *CaSOC1* were significantly higher in *CaFFN*-silenced plants than in the control (*P* < 0.01). In contrast, the expression level of *CaAGL15* in *CaFFN*-silenced plants was significantly lower than that in the control (*P* < 0.01) (Fig. 4b). The expression levels of microRNAs were determined relative to *CaU6* [*CA07g02150*]. *MiR172b* exhibited an increased level and *miR156e* represented a reduced level in *CaFFN*-silenced plants (Fig. 4b). The results suggested the role of CaFFN as a transcriptional activator to promote the expression of *CaAGL15* and *miR156e* and as a transcriptional repressor to repress the expression of *CaAG*, *CaAP1*, *CaSEP3*, *CaSOC1*, and *miR172b*.

### The CaFFN transcription factor directly activates the floral repressor gene *CaAGL15*

The finding that the expression level of the floral repressor gene *CaAGL15* was reduced in *CaFFN* gene-silenced pepper plants prompted us to examine whether *CaAGL15* is directly regulated by CaFFN. We checked the ability of CaFFN to bind to the cis-elements of *CaAGL15* using a yeast one-hybrid assay. The full-length cDNA of *CaFFN* was cloned in frame with the GAL4 activation domain (AD) in the pGADT7 vector. The cis-elements of *CaAGL15* were inserted into the pAbAi vector. The minimal inhibitory concentration of aureobasidin A (AbA) was 400 ng/ml for bait strains of *CaAGL15*. The results showed that CaFFN directly interacted with *CaAGL15*, since yeast transformed with pGADT7-CaFFN and the bait vector grew in SD/-Leu selection medium supplemented with the minimal inhibitory concentration of AbA, while yeast transformed with pGADT7-lam and pAbAi-P53, as a negative control, did not (Fig. 4c). In addition, a dual-luciferase assay was performed in *N*. *benthamiana* leaves. Recombinant pGREEN0800-*CaAGL15* containing the promoter region of *CaAGL15* (reporter) and the fused protein with CaFFN (effector) were coinfiltrated into leaves. Cotransformation of *CaAGL15* and the CaFFN protein resulted in a twofold LUC/REN ratio compared with the control (Fig. 4d). The results indicated that CaFFN can bind to the *CaAGL15* promoter in vivo and transactivate the expression of the reporter gene.

### *CaFFN* was significantly associated with flowering time in the pepper population

**Fig. 5:**
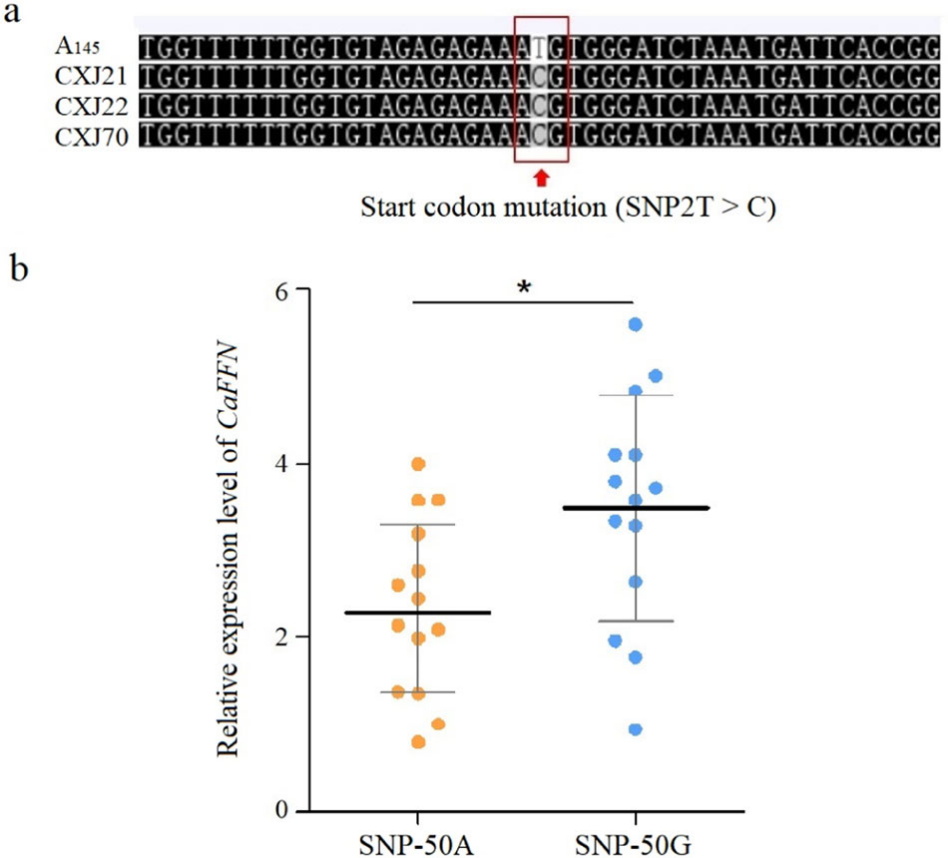
SNPs of the *CaFFN* gene in the pepper population. **a** Three additional pepper accessions were found to have the SNP2T > C mutation out of 383 pepper cultivars in the Pepper Pangenome Browser. **b** Relative expression level of *CaFFN* in the shoot apical meristem of 28 pepper accessions at the vegetative stage. Each data point represents the expression level of an accession. Black bars represent the median, and gray bars represent the interquartile range. Asterisks show significant differences between haplotypes SNP-50A and SNP-50G (Wilcoxon–Mann–Whitney test, *p < 0.05)

In this study, the *CaFFN* gene was shown to be a flowering repressor in pepper. To determine the distribution of the SNP2T > C mutation of the *CaFFN* gene in the pepper population, the *CaFFN* sequences of 383 pepper accessions were surveyed in PepperPan, a pepper pangenome browser^29^. The results showed that SNP2T > C was a rare SNP that occurred in only three additional accessions, CXJ21 (B33), CXJ22 (B35), and CXJ70 (A100) (Fig. 5a), and these three accessions were early flowering materials, with FFN numbers of 3, 3, and 5, respectively, suggesting that SNP2T > C could affect flowering time in multiple genetic backgrounds. The rare frequency of SNP2T > C also implies that the mutation occurred very recently and has not been widely used in breeding programs.

Due to the low number of accessions with nucleotide C at position 2, SNP2T > C could not be validated by association analysis. To further investigate whether *CaFFN* is an important regulator of flowering time in the pepper population, the numbers of FFNs in 164 pepper inbred lines were investigated. The numbers of FFNs exhibited wide phenotypic variations, ranging from 2 to 23, with an average of 10.5. Combined with the genotypic SNP/Indel data from the *CaFFN* sequenced region in 164 pepper accessions, an association analysis was performed to quantify significant associations. A SNP 50 bp upstream of the start codon (SNP-50G > A) was significantly associated with FFNs (*P* = 3.25E−07 < 0.01), explaining 14.5% of the phenotypic variation. The results demonstrated that *CaFFN* was significantly associated with flowering time in the pepper population.

A sequence comparison between the *CaFFN* alleles of 53 pepper accessions did not reveal clear causative polymorphisms in the coding region. It is speculated that the 5′-UTR may influence the number of FFNs by affecting the expression of the *CaFFN* gene. To test this hypothesis, 28 randomly selected accessions were divided into two haplotypes according to SNP-50G > A, and the expression level of the *CaFFN* gene was determined by qRT-PCR (Fig. 5b). Statistical analysis showed that SNP-50G > A significantly correlated with the expression level of the *CaFFN* gene (*P* < 0.05), suggesting that the expression level of *CaFFN* might be influenced by its 5′-UTR.

### Characterization of the *CaFFN/AP2* homologs in pepper

**Fig. 6:**
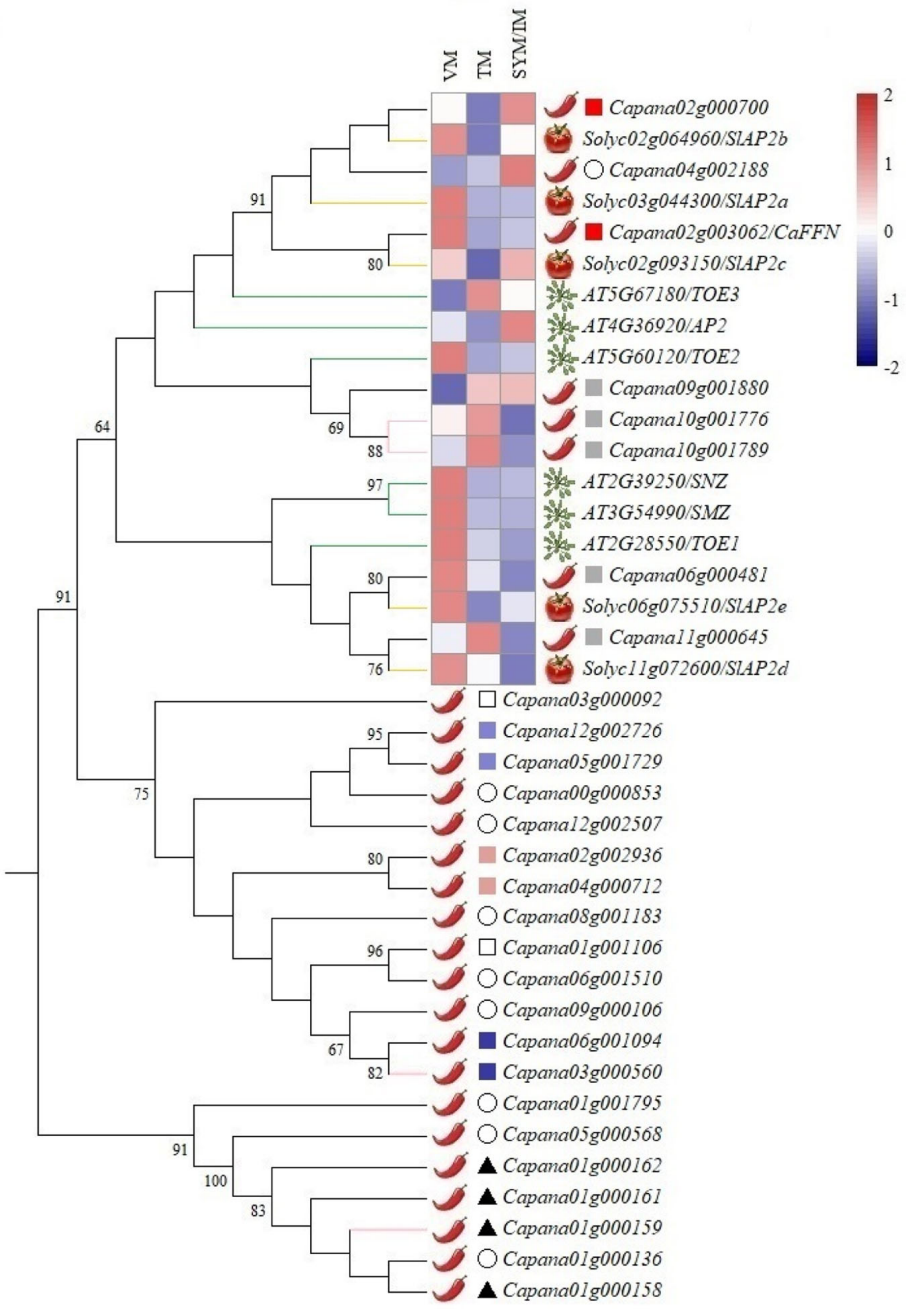
Phylogenetic tree and expression heatmap of *CaFFN*/*AP2* homologs. A phylogenetic tree was constructed by the maximum likelihood (ML) method; bootstrap values are based on 1000 replicates, and values lower than 60 are not shown; *AP2* family genes and related genes in pepper are indicated with black branches and pink branches; *CaFFN*/*AP2* homologs in tomato and *Arabidopsis* are indicated with yellow and green branches; dispersed, tandem, and whole-genome duplications/triplications (WGD/T) or segmental duplications are depicted by circles, triangles, and squares, respectively; duplicated gene pairs are indicated by squares in the same color, and retained genes with two copies of triplicated genes lost after triplication in pepper are indicated by hollow squares. An expression heatmap of 19 *CaFFN/AP2* homologs was constructed, and the color scale at the top right represents the expression values scaled by the Pheatmap package (https://CRAN.R-project.org/package = pheatmap) in R; the expression data of 19 *CaFFN*/*AP2* homologs were obtained from this study and previous studies^20,59,60^. VM represents the vegetative meristem, TM represents the transition meristem, SYM represents the sympodial meristem and IM represents the inflorescence meristem

CaFFN has two AP2 domains and is a member of the *AP2* family gene. *AP2* family genes with two or more AP2 domains play essential roles in plant growth and development^30–32^, as shown in this study. To systematically analyze the *AP2* family genes in pepper, the HMM profile with the AP2 domain (PF00847) was used as a query, and a total of 24 *AP2* family genes were obtained in the genome database of the cultivated pepper *Zunla-1* (Fig. 6). Duplication events are known to be the major driving force in the expansion of gene families^33–35^. To explore the major driving force for the expansion of the *AP2* family in pepper, the duplication types of each gene were detected by the MCScanX package, including singleton, dispersed, proximal, tandem and whole-genome duplication/triplication (WGD/T) or segmental duplication. Combined with the data of the retained triplicate genes from WGT in pepper^36^, 50.0% (12) of the pepper *AP2* family genes were assigned to WGD/T or segmental duplication types (Fig. 6). This indicated that WGD/T or segmental duplication was the major force for the expansion of the *AP2* family genes in pepper. Among them, the *CaFFN* (*Capana02g003062*) gene was located in a large collinear block containing 98 genes. The timing of this duplication event was estimated to be ~47.31 million years ago (Mya) based on the mean Ks value of segmental duplicated gene pairs within this collinear block. Thus, the segmental gene pair *CaFFN* and *Capana02g000700* may have arisen from a WGT before pepper-tomato divergence (20 Mya) and after pepper-grape divergence (89 Mya) with a copy of the triplicated gene lost^36^.

To compare the expression patterns of the *CaFFN*-related genes in pepper, *Arabidopsis* and tomato, the expression patterns of these genes in SAM at three different developmental stages were plotted in a heatmap (Fig. 6). Similar to the *CaFFN* gene, numerous *CaFFN*-related genes showed a relatively high expression level at the vegetative meristem stage and a low expression level at the transition meristem stage, except for *Capana04g002188*, *Capana09g001880*, *Capana10g001776*, *Capana10g001789*, *Capana11g000645*, and *TOE3*.

## Discussion

In this study, the locus controlling flowering time in the F_2_ population derived from an intraspecific cross between the early flowering pepper B_9431_ and the late-flowering pepper A_145_ was mapped to the end of chromosome 2. This locus was reported to be a flowering time locus in several previous reports^17,20,25^. Borovsky et al.^20^ provided supporting genetic evidence for the putative function of *CaAP2* in this chromosome region as a flowering repressor, but direct proof of the function of *CaAP2* is still lacking. The *CaAP2* gene (referred to in this study as the *CaFFN* gene) with a spontaneous natural point mutation (SNP2T > C) at the start codon in B_9431_ cosegregated with the FFN trait in the F_2_ population of 297 individuals; this is additional genetic evidence for the function of *CaFFN*/*CaAP2* as a flowering repressor. To provide more direct proof of the function of the *CaFFN* gene, transgenic *N. benthamiana* lines expressing the *CaFFN* gene were generated, and the flowering repressor function of this gene was verified by two *35S::CaFFNm3* transgenic lines with a high level of *CaFFN* mRNA that flowered two and a half months later than control plants. The function of the *CaFFN* gene was also verified by VIGS of the *CaFFN* gene in the pepper A_145_, which flowered earlier with a six-node reduction in the number of FFNs.

Putative *miR172* target sites in *AP2*-like genes are conserved in eudicots, monocots, and early land plants^37–39^, indicating that the expression of *AP2*-like genes could be regulated by *miR172*. Mlotshwa et al.^38^ generated transgenic *N. benthamiana* lines expressing *Arabidopsis* wild-type *AP2* (*35S::AP2*) and the *miR172*-resistant *AP2* mutant (*35S::AP2m3*). Nearly all *35S::AP2* plants were nonphenotypic with barely detectable levels of *AP2* mRNA; however, *35S::AP2m3* plants accumulated high levels of *AP2* mRNA and exhibited floral patterning defects such as the proliferation of numerous petals, stamens, and carpels. In our study, all *35S::CaFFN* plants were nonphenotypic with a low level of *CaFFN* mRNA. However, only two *35S::CaFFNm3* plants that accumulated high levels of *CaFFN* mRNA exhibited an obvious late-flowering phenotype and floral patterning defects. It is inferred that a visible flowering-time alteration in *N*. *benthamiana* requires *CaFFN* mRNA to reach a certain threshold. Further analyses are needed to clarify the reason for the different expression levels of *35S::CaFFNm3* plants. In contrast to *35S::AP2m3* plants with numerous petals, stamens, and carpels, these two *35S::CaFFNm3* plants exhibited short filaments, poorly developed petals, and a sterile phenotype. These results demonstrated that the *CaFFN* gene has a flowering repressor function as an *AP2* gene in *Arabidopsis* and likely results in differentiation in the floral patterning of pepper.

In *Arabidopsis*, *AP2* is involved in various developmental processes at the shoot apex^40,41^. The expression patterns of *AP2* and *CaFFN* in SAM were consistent with their function as flowering repressors, which showed a high expression level at the vegetative meristem stage and a low expression level at the transition meristem stage. Similar expression patterns were found for nine additional homologs in *Arabidopsis* and tomato, indicating that these genes may have similar/overlapping functions. Previous research has demonstrated that six AP2 domain-containing genes, including *AP2*, showed functional redundancy of their role in flowering time in *Arabidopsis*; the early flowering phenotype of the *ap2* mutant was largely obscured by the effects of the other five members of the eu*AP2* lineage^10^. In contrast, all of the *CaFFN* mutants in *C*. *annuum*, including E-62^20^, B_9431_, B33, B35, and A100, exhibited an obvious early flowering phenotype, which indicated that the flowering time control function of this gene had little redundancy with other genes in *C*. *annuum*. This result was further confirmed by the expression patterns of *CaFFN/AP2* homologs in the SAM of *C*. *annuum*, which differed from that of most of the detected *CaFFN/AP2* homologs (Fig. 6), representing a functional differentiation of these homologs. On the other hand, no floral patterning defects were discovered in E-62, B_9431_, B33, B35, and A100, indicating that the function of floral patterning regulation of the *CaFFN* gene was redundant and could be compensated by other gene(s) in pepper.

*Capana02g000700*, as a paralog of *CaFFN* that has arisen from an ancient WGT (~47.31 Mya), might have some functional redundancy with *CaFFN* since it presented a similar expression pattern as *CaFFN* in the SAM of pepper (Fig. 6). The possible function of *Capana02g000700* in flower development and transcriptional repression of flowering time was also reported in a previous study^26^. However, *Capana02g000700* may only have a weak effect on flowering repressor in *C*. *annuum*, since its expression level in the early flowering inbred line CA1 (*C*. *annuum*) was much lower than that in the late flowering inbred line 740 (*C*. *chinense*)^26^. This is a reasonable explanation for why the early flowering phenotype of the *CaFFN* mutants in *C*. *annuum* was not obscured by *Capana02g000700*.

To investigate how the *CaFFN* gene participates in the complicated network to affect flowering time and floral organ morphogenesis, the transcription profiles of several key flowering time and organ development genes were assayed by qRT-PCR. The results suggested that CaFFN controls flowering time and floral development by regulating genes that are involved in the aging pathway (Fig. 4b): CaFFN functions as a transcriptional repressor to suppress the expression of the floral activator genes *CaAP1*, *CaSEP3*, and *CaSOC1* as well as the key floral identity gene *CaAG*; it also functions as a transcriptional activator to activate the expression of the floral repressor gene *CaAGL15*. In addition, CaFFN negatively regulated *miR172* and positively regulated *miR156*, as indicated by their expression relationship. Moreover, yeast one-hybrid assays and dual-luciferase reporter assays revealed that CaFFN directly activates the floral repressor gene *CaAGL15*. These results provide the first insight into the mechanisms employed by CaFFN to regulate flowering time and floral patterning in pepper.

Genome-wide association analysis (GWAS) of 36 agronomic traits, including the FFN trait, in 287 pepper accessions was carried out based on SLAF-seq data, but no peak regions associated with the FFN trait were detected^22^. It is unclear whether the *CaFFN* gene has a broad effect on flowering time in the pepper population. In our study, *CaFFN* gene association analysis was performed in 167 pepper inbred lines. A SNP 50 bp upstream of the start codon was significantly associated with the number of FFNs, explaining 14.5% of the phenotypic variation. This result indicated that the *CaFFN* gene has a broad effect on flowering time in the pepper population. This broad effect is not related to the SNP2T > C mutation at the start codon of *CaFFN*, which is a rare mutation in the pepper population, but is most likely due to the variation(s) in the 5′-UTR of the *CaFFN* gene. In addition to the result that the SNP in the 5′-UTR (SNP-50G > A) of *CaFFN* was significantly associated with the FFN trait, the results that SNP-50G > A significantly correlated with the expression level of the *CaFFN* gene indicated by qRT-PCR also support the speculation that the 5′-UTR may influence the number of FFNs by affecting the expression of the *CaFFN* gene in the pepper population. Whether SNP-50G > A is the causative mutation that influences the expression of *CaFFN* or a SNP tightly linked with the causative mutation needs further research.

The control of flowering time in pepper is an attractive objective for breeding aimed at creating locally adapted cultivars. In pepper, several genes, including *CaJ*, *CaBL*, *CaS*, *FA*, and *CaAP2*^14,15,19–21^, were reported to be flowering regulators. Only *FA* and *CaAP2*/*CaFFN* were described as flowering repressor genes, and the effect of the *FA* gene on flowering time was minor^20,21^. Despite a certain degree of reduction in shoot and fruit size and the extreme flowering time phenotype in the homozygous mutant of the *CaFFN* gene^20^, it is still an ideal breeding material for genetic improvement. Mutated *CaFFN* could significantly reduce the number of FFNs with minimal negative impact on yield in multiple genetic backgrounds when heterozygous, making it very useful for hybrid pepper breeding. This gene was used to select early maturing pepper varieties in our pepper breeding experiments.

## Materials and methods

### Plant materials

The precocious pepper B_9431_ was crossed with the late-flowering inbred line A_145_ (from the Asian Vegetable Research and Development Center). The F_1_ hybrid was selfed to generate a segregating F_2_ population. A total of 297 individuals of the F_2_ population were used for mapping. The flowering time of pepper was measured by counting the number of leaves on the primary stem between the cotyledon and first flower. All plants were grown in a greenhouse in Nanchang (28°33′N, 115°56′E, China).

### BSR-seq

BSR-Seq combined bulked-segregant analysis and RNA-Seq and was performed to map loci for target traits. For the genetic analysis, two pools were constructed; each pool contained 30 individuals from the F_2_ segregating population with extreme phenotypes. For the phenotyping of the FFN trait, total RNA of leaves at the blooming period was extracted using RNAiso Plus reagent (Takara, Dalian, China). RNA was quantified and assessed using a Qubit Fluorometer (Invitrogen, Carlsbad, USA), Nanodrop spectrophotometer (Thermo Fisher Scientific, Wilmington, USA) and Agilent 2100 bioanalyzer (Agilent Technologies, Palo Alto, USA). Paired-end sequencing was carried out on an Illumina HiSeq X-ten instrument. Clean RNA-seq data were mapped to the *C*. *annuum* cv. CM334 genome chromosomes (release 1.55, http://peppergenome.snu.ac.kr/)^42^ using BWA software^43^. SNPs were screened using SAM tools^44^. The differences in allele frequencies of the 12 pepper chromosomes between the two pools were calculated and plotted.

### SNP marker development and genotyping

To develop a SNP marker for the *CaFFN* gene, which was speculated to be the candidate gene controlling flowering time in pepper, the genomic sequences of the *CaFFN* gene in B_9431_ and A_145_ were amplified (with primers listed in Supplementary Table S1) and sequenced. The polymorphic SNP (SNP2T > C) at position 2 of the *CaFFN* gene between the B_9431_ and A_145_ genotypes was converted into the CAPS marker CSF2 for genotyping. PCR was carried out in B_9431_, A_145_, the F_1_ population, and the F_2_ population (297 individuals), and each reaction had a total volume of 10 μl containing 50 ng genomic DNA, 0.2 μM primer FFN1-5UF4, 0.2 μM primer FFN1-e1R2 (Supplementary Table S1) and 5 μl 2×Taq Master Mix (Novoprotein Scientific Inc., Shanghai, China). PCR was performed with the following conditions: denaturing for 1.5 min at 94 °C, followed by 38 cycles of at 94 °C for 20 s, 55 °C for 20 s, and 72 °C for 1 min, and a final elongation at 72 °C for 5 min. The amplified PCR products were digested with the restriction enzyme *TaiI* (Thermo Fisher Scientific, Waltham, USA) directly in a final volume of 10 μl consisting of 0.65 μl 10 × R Buffer, 3.5 μl PCR product, 0.2 μl *TaiI* and 5.65 μl ddH_2_O. The mixture was incubated at 65 °C for 15 h and 80 °C for 20 min and subjected to gel electrophoresis on a 2% agarose gel.

### Plant transformation of *N. benthamiana*

*CaFFN* gene overexpression vectors were generated by cloning into the *XhoI* + *XbaI* digestion sites of the vector pHellsgate 8. In this study, two different overexpression vectors were constructed: one included the wild-type *CaFFN* cDNA of A_145_ (35S::*CaFFN*), and the other included mutant *CaFFN* cDNA with six mismatches to *miR172* that did not change the amino acid sequence (synonymous mutations) (35S::*CaFFNm3*). The recombinant plasmid was introduced into *N. benthamiana* by *Agrobacterium-*mediated transformation as described previously^45^. The plants were screened in medium supplemented with 75 mg/L kanamycin (the negative control could not regenerate) in a plant growth chamber at 24 °C under 16 h light/8 h dark cycles. The transgenic seedlings were identified by the primers FFN-RT-F and HR-gate8-RV (Supplementary Table S1).

### VIGS in pepper A_145_

For *CaFFN* silencing analysis, the specific coding regions of the *CaFFN* gene were used for VIGS vector construction. The sequence specificity was confirmed by genome-wide homologous sequence searching by BLAST against sequences in the Zunla databases (http://peppersequence.genomics.cn/page/species/blast.jsp). Two fragments of ~200 bp in different regions of the *CaFFN* gene were inserted into the pTRV2 vector at the *EcoRI* site to construct two vectors, TRV2::*CaFFN-*1 and TRV2::*CaFFN*-2. In addition, a fragment of ~100 bp of the *CaPDS* gene was inserted into pTRV2 to construct a positive control vector, TRV2::*CaPDS*. The resulting vectors TRV2::*rec* and TRV1 were transformed into *A*. *tumefaciens* (strain GV3101). *A*. *tumefaciens* cells harboring TRV1 and TRV2::*CaFFN*-1/TRV2::*CaFFN*-2, TRV2 (as a negative control) or TRV2::*CaPDS*, as a positive control (resuspended in induction medium at a 1:1 ratio, OD_600_ = 0.6), were coinfiltrated into seedlings of pepper A_145_ at the four-leaf stage. The infiltrated seedlings were grown in a phytotron in the dark for 24 h and then at 20 ± 1 °C under 16 h light/8 h dark cycles with a relative humidity of ~90 ± 5%.

### Quantitative reverse transcription PCR

To determine the relative transcription levels of selected genes, total RNA was extracted from plant tissue using RNAiso Plus reagent (Takara, Dalian, China) according to the manufacturers’ instructions. Single-stranded cDNA was synthesized using TransScript^®^ II One-Step gDNA Removal and cDNA Synthesis SuperMix (TransGen Biotech., Beijing, China) for quantitative reverse transcription PCR (qRT-PCR) analysis of target genes or using TransScript^®^ miRNA First-Strand cDNA Synthesis SuperMix (TransGen Biotech., Beijing, China) for qRT-PCR analysis of target microRNAs. Quantitative real-time PCR was performed on a Bio-Rad CFX Connect Real-Time System (Bio-Rad Laboratories, Hercules, USA) using NovoStart^®^ SYBR qPCR SuperMix Plus (Novoprotein Scientific Inc., Shanghai, China). Gene expression was calculated relative to *CaGAPDH* or *CaU6* in pepper or relative to *NbbTUB* in tobacco by the 2^−ΔΔCT^ method^46^. The amplification program was performed at 95 °C for 2 min, followed by 95 °C for 10 s and 60 °C for 30 s (45 cycles). The primers used for qRT-PCR are listed in Supplementary Table S1. In each case, three technical replications were performed for each of at least three independent biological replicates.

### Yeast one-hybrid (Y1H)

To investigate the interaction between the CaFFN transcription factor and *CaAGL15*, the full-length *CaFFN* open reading frame (ORF) sequence was transformed into the pGADT7 vector to provide the prey protein, and the cis-elements (~2000 bp upstream of the start codon isolated from pepper A_145_) of the *CaAGL15* gene were inserted into the pAbAi vector as bait sequences. The linearized bait plasmid pAbAi-*CaAGL15* was transformed into the Y1H Gold yeast strain, and the minimal inhibitory concentration of AbA was determined for the bait strain with the empty pGADT7 vector. Then, the prey plasmid pGADT7-CaFFN was transformed into the bait strain to detect the DNA-protein interaction by incubating them at 30 °C for 3 days on SD/-Leu medium supplemented with the minimal inhibitory concentration of AbA.

### Dual-luciferase reporter assay

The cis-element of *CaAGL15* (~2000 bp upstream of the start codon isolated from pepper A_145_) was inserted into pGREEN0800-LUC^47^ to generate the reporter plasmid pGreen0800-*CaAGL15*. The full-length ORF of *CaFFN* was inserted into the pGreenII 62-SK vector to generate the effector vector. The effector and reporter *Agrobacterium* cultures were mixed together and infiltrated into *N*. *benthamiana* leaves^48^. Empty pGreenII 62-SK (EV) was cotransformed with the *CaAGL15* reporter as a no-interaction control. Firefly luciferase and Renilla luciferase were quantified at 3 days after infiltration using Dual Luciferase Reporter Assay System reagents (Promega, Fitchburg, United States). Three biological replicates were conducted for each combination.

### Association analysis of *CaFFN* genes

A forward primer FFN-solcs-5UF1 and a reverse primer FFN-solcs-e4R1 (Supplementary Table S1) were used to amplify a fragment of the *CaFFN* gene in 164 inbred lines from core pepper collections. Each amplified fragment was sequenced using the FFN-solcs-5UF1 primer, and the sequences obtained were aligned using MUSCLE^49^. SNPs and InDels were identified, and variants were filtered according to the MAF (MAF ≥ 5%). The variants from 164 pepper inbred lines and FFN phenotypes were used in the association analysis, which was conducted using the cMLM in TASSEL v3.0^50^. The population structure (Q matrix) and familial kinship (K matrix) derived from our previous studies^51^ were taken into account to avoid spurious associations. The associated variant(s) with the FFN trait were identified at the probability level of 0.01.

### Analysis of *AP2* family genes

The genome database of the cultivated pepper *Zunla-1*^36^ was used for the identification of *AP2* family genes. The hidden Markov model (HMM) profile of the AP2 domain (Pfam Accession No. PF00847) was used as a query to BLAST against the *Zunla-1* protein database. AP2 family members were identified by checking gene structures in Pfam^52^ to confirm the presence of two or more AP2 domains^31,53^. BLASTP alignment (*E* < 1e−5, top five matches) was carried out across the whole-genome protein sequences, and the output results were loaded into MCScanX (MATCH_SIZE: 5)^54^ to identify collinear blocks and to distinguish the singleton, dispersed, proximal, tandem, and WGD/segmental duplication types of these genes. The coding sequences of the *AP2* family genes and four additional genes (*Capana01g000159*, *Capana10g001789*, *Capana03g000560*, and *Capana10g001776*) were used to create a codon alignment by DAMEB6^55^. A phylogenetic tree of *CaFFN/AP2* homologs was constructed using the maximum likelihood (ML) method in MEGA6^56^, and branch reliability was assessed with 1000 bootstrap replications. The Ks (synonymous substitutions per site) values were calculated in PAML^57^. The formula T = Ks/2 R was used to estimate the divergence time, where R is 1.5 × 10^−8^ synonymous substitutions per site per year^58^. The expression data of 19 *CaFFN/AP2* homologs were obtained from this study (primers are listed in Supplementary Table S1) and previous studies^20,59,60^. The expression data were scaled to construct the heatmap by the Pheatmap package (https://CRAN.R-project.org/package=pheatmap) in R.

## Supplementary information

Supplementary Table S1 Primers used in this study
