# Supplementary material for: The APETALA2 homolog CaFFN regulates flowering time in pepper
Source: Hortic Res. 2021 Nov 1;8:208. doi: 10.1038/s41438-021-00643-7 (PMC8558333; doi:10.1038/s41438-021-00643-7)
Supplement: Supplementary file 1 — Supplementary Table S1 Primers used in this study [file 41438_2021_643_MOESM1_ESM.doc]

| Supplementary Table S1 Primers used in this study | |  |
| --- | --- | --- |
| Primer | Primer sequence (5'-3') | Purpose |
| FFN1-5UF4 | TAAAAGTGGCCTAGTCCATGTCTG | *TaiI* digestion, primers for the CAPS marker CSF2 |
| FFN1-e1R2 | GCCATATCTATAGGCTTAGCCAATC |
| CaFFN-ATG-F | CATTTGGAGAGGACACGCTCGAGATGTGGGATCTAAATGATTCACCG | Primers for amplification of the CDS of the *CaFFN* gene to construct the *35S::CaFFN* vector, including adaptors for vector pHellsgate 8 (*XhoI*+*XbaI* digested) ligation |
| CaFFN-TGA-R | TCTCATTAAAGCAGGACTCTAGATCAAGATGGTCTCATGAGTGAGTGAT |
| FFN-miR-F | CTGCAGCTGCTTCCTCTGGTTTCCCCCAGCAGATACCAAG | Primers for introduction of 6 mismatchs into the miR172 recognition site of the *CaFFN* gene to construct the *35S::CaFFNm3* vector |
| FFN-miR-R | CTGGGGGAAACCAGAGGAAGCAGCTGCAGCTGCAAATATTTGAGGAG |
| FFN-RT-F | TCAGATTCAATACCCAAGCGGC | Primers for detection of *35S::CaFFN* and *35S::CaFFNm3* transgenic *Nicotiana benthamiana* plants |
| HR-gate8-RV | CGGTAAGGATCTGAGCTACACAT |
| CaFFN-spVG-F1 | TGAGTAAGGTTACCGAATTCCACCGGATCGACGAAGGC | Primers for amplification of the specific region of the *CaFFN* gene to construct the TRV2::*CaFFN*-1 vector, including adaptors for vector pTRV2 (*EcoRI* digested) ligation |
| CaFFN-spVG-R1 | GGAGGCCTTCTAGAGAATTCGGCTTTTCTTCTTCTTCATCATCAG |
| CaFFN-spVG-F2 | TGAGTAAGGTTACCGAATTCCTGATGATGAAGAAGAAGAAAAGCC | Primers for amplification of the specific region of the *CaFFN* gene to construct the TRV2::*CaFFN*-2 vector, including adaptors for vector pTRV2 (*EcoRI* digested) ligation |
| CaFFN-spVG-R2 | GGAGGCCTTCTAGAGAATTCGCTGCTGCTGCTGTTGCTG |
| Sol-PDS-csF1 | TGAGTAAGGTTACCGAATTCCAAATTGGACTTGTTTCTGCTG | Primers for amplification of the *CaPDS* gene to construct the TRV2::*PDS* vector, including adaptors for the vector pTRV2 (*EcoRI* digested) ligation |
| Sol-PDS-csR1 | GGAGGCCTTCTAGAGAATTCTTCAAACCAAACCTTTAAAGGC |
| bTUB-qF2 | AYGCTGATGAATGTATGGTCCTTGA | QRT-PCR primers for the *NbbTUB* gene in *N*. *benthamiana* |
| bTUB-qR2 | GCATCCCACATTTGTTGAGTAAGC |
| FFN-RT-jF4 | CGAAAATGAACTGAACTCACTTGAATCT | QRT-PCR primers for the *CaFFN* gene in transgenic *N. benthamiana* |
| CaFFN-sNR2 | TTCGTGCATCGATAAGGGC |
| GAPDH-qF3 | GGCTGCCATTAAGGAGGAGTCTG | QRT-PCR primers for the *CaGAPDH* gene in pepper |
| GAPDH-qR3 | ACCACTCGCGTGCTGTAACCC |
| CaU6 | GACATCCGATAAAATTGGAACGA | QRT-PCR forward primer for the *CaU6* gene in pepper |
| FFN-RT-jF4 | CGAAAATGAACTGAACTCACTTGAATCT | QRT-PCR primers for the *CaFFN* gene in pepper |
| FFN-RT-R4 | CTTCATTGTTCCCACTATCTCTTCTTC |
| CaAG-qF6_2 | GATTGTCTTCTCAAGCAGAGGCAG | QRT-PCR primers for the *CaAG* gene in pepper |
| CaAG-qR6 | CTTTCACACTGTTGTTGGCATACTC |
| CaAGL15-qF5 | GAAGAGCTCCGAGGGTTATATC | QRT-PCR primers for the *CaAGL15* gene in pepper |
| CaAGL15-qR1 | GGATGATGGTATTCAAGATAGGCTG |
| CaAP1-qF5_2 | GAAGACACAACCCAACTCAGGC | QRT-PCR primers for the *CaAP1* gene in pepper |
| CaAP1-qR1 | CATCTATGTTCATTTCCCTTGTTCC |
| CaSEP3-qF5_2 | CGTTACGAGGCACTACAGCGA | QRT-PCR primers for the *CaSEP3* gene in pepper |
| CaSEP3-qR5_2 | ATCCAACATCAACTGAGTCCGA |
| CaSOC1-qF1 | AAGATTGAAAGAAAAGGAGAAAACC | QRT-PCR primers for the *CaSOC1* gene in pepper |
| CaSOC1-qR1 | TCTGATTTATCGCTACCACCACC |
| PmiR156e-F | CCCTGACAGAAGAGAGTGAGCAC | QRT-PCR forward primer for *miR156e* in pepper |
| PmiR172b-F | GGAGAATCTTGATGATGCTGCAT | QRT-PCR forward primer for *miR172b* in pepper |
| Universal-qR | GATCGCCCTTCTACGTCGTAT | QRT-PCR reverse primer for CaU6, miR156e and miR172b in pepper |
| Ca2g700-qF1 | TGAGCCAAACACACCTGCATTC | QRT-PCR primers for the *Capana02g000700* gene in pepper |
| Ca2g700-qR3 | CCATTGATCTCTTCTATTGCTGCTG |
| Ca4g2188-qF2 | AGTGCAAAGGTTTGGCCAATATG | QRT-PCR primers for the *Capana04g002188* gene in pepper |
| Ca4g2188-qR3 | CTTGTTGCCCTAATTTGGCCTTC |
| Ca6g481-qF1 | AAGCTCAATCGTGGACTGGAATC | QRT-PCR primers for the *Capana06g000481* gene in pepper |
| Ca6g481-qR1 | AACCTGGCTGGGCATTTGC |
| Ca9g1880-qF3 | TTCTACAGAAATGGGAAGCAAACT | QRT-PCR primers for the *Capana09g001880* gene in pepper |
| Ca9g1880-qR2 | CTGTTGTTGAATTAGCGAATCCTG |
| Ca10g1776-qF3 | GTGCACATACTTCGACGTCATAGC | QRT-PCR primers for the *Capana10g001776* gene in pepper |
| Ca10g1776-qR1 | GTGATCGTCTCCCTTCCATTGC |
| Ca10g1789-qF2 | GACAATCAACATGGAAAAACTACCC | QRT-PCR primers for the *Capana10g001789* gene in pepper |
| Ca10g1789-qR1 | AGAGGTGTCAACTTCCAAGCCC |
| Ca11g645-qF2 | CAAGGGGCTACCTGCAGTCAC | QRT-PCR primers for the *Capana11g000645* gene in pepper |
| Ca11g645-qR2 | GGTTGTTCCGACTTGACCATGC |
| FFN-solcs-5UF1 | GSTAGTATTCTAAAAGTGGGCTAGTCC | Primers for amplification of the *CaFFN* gene fragment in 164 pepper inbred lines |
| FFN-solcs-e4R1 | CCTCTAAGCTAAAGTTTATGTCSGC |
| FFN-solcs-5UF1 | GSTAGTATTCTAAAAGTGGGCTAGTCC | Primers for amplification of genomic sequence of the *CaFFN* gene in B_9431_ |
| FFN-solcs-e4R1 | CCTCTAAGCTAAAGTTTATGTCSGC |
| FFN-csF5 | GTGGATTTGATACTGCACATGC | Primers for amplification of genomic sequence of the *CaFFN* gene in B_9431_ |
| FFN-seqR2 | ACTGCATCCTTCCCGTTACAC |
| FFN-csF6 | ACACAAATGTGGTAGATGGGAAGC | Primers for amplification of genomic sequence of the *CaFFN* gene in B_9431_ |
| FFN-RT-R2 | GTGTGTCTGGCTCAATAGTTGCAAGG |
| FFN-RT-F2 | CGAATTGGGGGATAATAGAGGAGG | Primers for amplification of genomic sequence of the *CaFFN* gene in B_9431_ |
| FFN-3R2 | GAATGGGGCAAATTGGCAS |
| FFN-seqF1 | CAGTACCGTGGGGTTACCTTCTA | Primers for amplification of genomic sequence of the *CaFFN* gene in A_145_ |
| CaFFN-terLA-R2 | TCTTGCCTTCAGTTAGTATTGGAGT |
| CaFFN-ProLA-F1 | CGACTCCATATTCTTTGTTAAACCTT | Primers for amplification of genomic sequence of the *CaFFN* gene in A_145_ |
| FFN-seqR2 | ACTGCATCCTTCCCGTTACAC |
